# Identifying Dynamic Patterns of Polypharmacy for Patients with Dementia from Primary Care Electronic Health Records: A Machine Learning Driven Longitudinal Study

**DOI:** 10.14336/AD.2022.0829

**Published:** 2023-04-01

**Authors:** Elisabetta Longo, Bruce Burnett, Sarah Bauermeister, Shang-Ming Zhou

**Affiliations:** ^1^The Aptuit, an Evotec company, Via Alessandro Fleming 4, 37135 Verona, Italy.; ^2^The Institute of Life Science, Swansea University Medical School, Swansea, SA2 8PP, UK.; ^3^Dementias Platform UK, Department of Psychiatry, University of Oxford, Oxford OX3 7JX, UK; ^4^The Faculty of Health, University of Plymouth, Plymouth, PL4 8AA, UK.

**Keywords:** dementia, polypharmacy, patient safety, diagnosis, electronic health records, machine learning, exploratory factor analysis

## Abstract

It is unclear how medication use evolved before diagnosis of dementia (DoD). This study aims to identify varied patterns of polypharmacy before DoD, their prevalence and possible complications. We collected primary care e-health records for 33,451 dementia patients in Wales from 1990 to 2015. The medication uses in every 5-year period along with 20-years prior to dementia diagnosis were considered. Exploratory factor analysis was used to identify clusters of medicines for every 5-year period. The prevalence of patients taking three or more medications was 82.16%, 69.7%, 41.1% and 5.5% in the Period 1 (0-5 years before DoD) ~ Period 4 (16-20 years before DoD) respectively. The Period 1 showed 3 clusters of polypharmacy - medicines for respiratory/urinary infections, arthropathies and rheumatism, and cardio-vascular disease (CVD) (66.55%); medicines for infections, arthropathies and rheumatism (AR), cardio-metabolic disease (CMD) and depression (22.02%); and medicines for arthropathies, rheumatism and osteoarthritis (2.6%). The Period 2 showed 4 clusters of polypharmacy - medicines for infections, arthropathies, and CVD (69.7%); medicines for CVD and depression (3%); medicines for CMD and arthropathies (0.3%); and medicines for AR, and CVD (2,5%). The Period 3 showed 6 clusters of polypharmacy - medicines for infections, arthropathies, and CVD (41.1%); medicines for CVD, acute-respiratory-infection (ARI), and arthropathies (1.25%); medicines for AR (1.16%); medicines for depression, anxiety (0.06%); medicines for CMD (1.4%); and medicines for dermatologic disorders (0.9%). The Period 4 showed 3 main clusters of polypharmacy - medicines for infections, arthropathy, and CVD (5.5%); medicines for anxiety, ARI (2.4%); and medicines for ARI and CVD (2.1%). As the development towards dementia progressed, the associative diseases tended to cluster with a larger prevalence in each cluster. Farther away before DoD, the clusters of polypharmacy tended to be clearly distinct between each other, resulting in an increasing number of patterns, but in a smaller prevalence.

Dementia is a progressive, chronic, and incurable neurodegenerative disorder seen most commonly in older people and characterized by a decline in cognitive performance which results in suffering and loss as patients develop impairments in memory, judgment, language, behaviour, and function. More than 46.8 million people worldwide were diagnosed with dementia and 2.5 million new cases are forecast to be diagnosed every year until 2050, impacting on direct and indirect healthcare costs [1, 2]. The symptoms and complications characterising dementia are severe and diverse, such as memory loss, disorientation, malnutrition, dehydration, falls [3], delirium, depression and other behavioural disorders [4]. A variety of medications are often prescribed for people with dementia (PwD) to manage such symptoms, dramatically increasing the risk of polypharmacy.

Polypharmacy is often driven by the introduction of multiple preventative medicines aiming to reduce the risk of future morbidity and mortality in specific health conditions. The World Health Organisation (WHO) Third Global Patient Safety Challenge [5] has treated the appropriate management of polypharmacy as a key flagship challenge to address, aiming to reduce severe, avoidable, medication-related harm by 50% over 5 years, globally. Polypharmacy is generally associated with the presence of multimorbidity in the same individual, but the causal link between the two is unclear, especially when dealing with elderly people [6]. With the introduction of multiple preventative medicines, the absolute benefit made by each additional medicine is likely to reduce; often referred to as the “law of diminishing returns” [7]. If the established criteria to optimise the use of medicines in individuals with multiple conditions are not followed, the medications may increase the risk of harm from drug interactions or side effects. The larger number of drugs is found to be positively associated with a higher likelihood of hospital re-admission within 3-month after hospital discharge [8]. Potential drug-drug interactions frequently lead to a more than twofold the increased risk of mortality within 3-month post-discharge [9]. Additionally, drugs with anticholinergic effects in polypharmacy are associated with an increased risk of cognitive and functional impairment, falls and all-cause mortality in older people [10, 11]. Using UK general practice data from the Clinical Practice Research Datalink (CPRD), a large, nested, case-control study found the significant associations between some classes of anticholinergic medicines and an increase in incidence of dementia [12]. Inappropriate polypharmacy [13], therefore, increases the risk of emergency department visits, drug-drug interaction, drug-disease interaction and eventually death [14].

Conversely, the risk of harms is likely to increase more medicines a person takes. Park et al. identified hypertension, diabetes, mental disorders, chronic liver, obstructive pulmonary diseases and congestive heart failure as highly correlated with polypharmacy [2]. Multiple medication use is very frequent in elderly people affected by multimorbidity, i.e. the occurrence of multiple and coexistent chronic diseases in the same individual [15] and presents an ongoing clinical challenge [16]. Particularly, where multiple comorbidities are present, PwD are more likely to be affected by polypharmacy even after adjusting for confounding factors such as age and gender [17, 18]. An emerging evidence showed that one year from the diagnosis, the total number of prescribed drugs increased by 10% in those with Alzheimer's disease and 15% in those with Lewy body dementia [19]. PwD have a higher burden of comorbid physical disease and polypharmacy than those without dementia.

Health organisations have indicated to minimise the use of medicines associated with increased anti-cholinergic burden, and if possible look for alternatives [20]. However, there is insufficient evidence to recommend one over the others. Health professionals recognise that optimising a person's medicines can support the management of long-term health conditions, multimorbidity and polypharmacy [21]. But medicine optimisation needs a deep understanding of patterns of polypharmacy and multimorbidity in the population of interested disease. Navigating medication management for PwD remains a challenge for clinicians [22]. Most studies on polypharmacy for PwD focus on cohorts with an established DoD [23, 24]. It is unclear how the medication use varied and evolved towards the DoD. Investigating the concomitant use and common prescription patterns of major drugs before the DoD could provide insights into the impacts of polypharmacy on development of dementia, or opportunities for early intervention in prescribing, and support the identification of medications at risk of drug-drug interactions.

The objectives of this study are: 1) to identify medicines that constitute the patterns of polypharmacy before the DoD; 2) to estimate the prevalence of these patterns; and 3) to gain understanding of how the patterns of polypharmacy vary before the DoD. This study uses exploratory factor analysis (EFA), a machine learning technique. This technique provides an effective tool to discover patterns without formulating precise hypotheses on the model built or on the data. Recognising and observing the variation in the patterns may provide insights on a causal link between polypharmacy and dementia diagnosis.

## MATERIALS AND METHODS

### Data Extraction

A retrospective longitudinal study was conducted to identify 33,451 dementia patients from more than 42 million row electronic GP records in Wales from 1990 to 2015 via the Secure Anonymised Information Linkage (SAIL) Databank [25] for identifying the cohort of PwD by the previously validated phenotypes [26]. All the Read codes regarding medications and diagnoses, together with dates of birth and gender information before DoD were extracted from the GP data. But the medication Read code dataset with dates of birth and gender was separated from the diagnostic code dataset while both datasets shared the same encrypted patient identifier.

In the first step, only the medication dataset was used to identify the patterns of medications. In this study, polypharmacy is considered as more than three prescribed medicines taken by one individual [27]. Aging is a risk factor for dementia because dementia can take a long time to develop. A person's risk then increases as they age, roughly doubling every five years [28, 29]. In order to assess the variation of patterns of polypharmacy, the dataset was split into four time-periods with five years each - Period 1: from 0 to 5 years before the DoD; Period 2: from 6 to 10 years before the DoD; Period 3: from 11 to 15 years before the DoD; Period 4: from 16 to 20 years before the DoD. For each time-period, the number of medicines prescribed was used to identify the clusters of medications independent from the comorbidities recorded for each individual.

Once the clusters of medications were identified, the second step was to merge these identified clusters with the diagnosis dataset, using the person-based identifier as a key, then calculate the prevalence of medications in each cluster. Each cluster was then labelled according to the diseases the medications in the cluster were prescribed for.

### Data Analysis

The large number of medication codes in GP data leads to the datasets with extremely high dimensions. To effectively analyse the high dimensional data and identify the natural clusters of the data fields in an integrative manner, the EFA was used to identify the underlying relations between medications without prior hypotheses on the data through the factor loadings which are measures of influence of the common factors on the variables under analysis [30]. EFA aims at reducing the number of medications into compact sets, also called factors [31], which can explain the variations in the data. EFA allows for the medications to be included in more than one factor (pattern) [32]. As such, EFA can effectively uncover the underlying structure of a relatively large set of medications. A parallel analysis was used to determine the optimal number of factors [33]. A factor loading over 0.30 was considered meaningful [33]. A maximum a posteriori (MAP) estimation was used to derive the factor scores for each individual patient.

Once the latent factors describing the underlying structures of medications were identified, we calculated the proportion of patients included in each of them. In this study, we estimated the number of patients belonging to a specific cluster by including an individual into the factor if at least three drugs inside it were prescribed to that individual.

The data was extracted using SQL on the SAIL Databank server, and the data analysis was conducted using R version R 3.5.0 [34].

### Ethics

Because the SAIL databank holds the patient records which have been anonymised and granted with the permission of relevant Caldicott Guardian/Data Protection Officer, no ethical review was required. However, an approval to proceed with the study was given by the Information Governance Review Panel [35].

**Table 1:** Gender and age composition in Period 1.

Gender composition			
	CLUSTER 1	CLUSTER 2	CLUSTER 3
MALES	6335 (35.30%)	2084 (35.10%)	167 (23.80%)
FEMALES	11612 (64.70%)	3854 (64.90%)	534 (76.20%)
Age composition			
AGE BAND	CLUSTER 1	CLUSTER 2	CLUSTER 3
<65	998 (5.56%)	333 (5.60%)	25 (3.54%)
65-75	2441 (13.60%)	808 (13.60%)	61 (8.66%)
75-85	7778 (43.34%)	2565 (43.20%)	278 (39.60%)
>86	6730 (37.50%)	2233 (37.60%)	338 (48.20%)

## RESULTS

Females accounted for 65.8% of the 33,451 PwD. The average age at diagnosis was 72.75 years with 82.2 years for females and 66.2 years for males. For each of the four 5-year periods, the number of identified clusters and their compositions varied. Particularly, the prevalence of patients taking three or more medication in different periods changed dramatically, which were 82.16%, 69.7%, 41.1% and 5.5% in the Periods 1~4 respectively.

**Figure 1.**
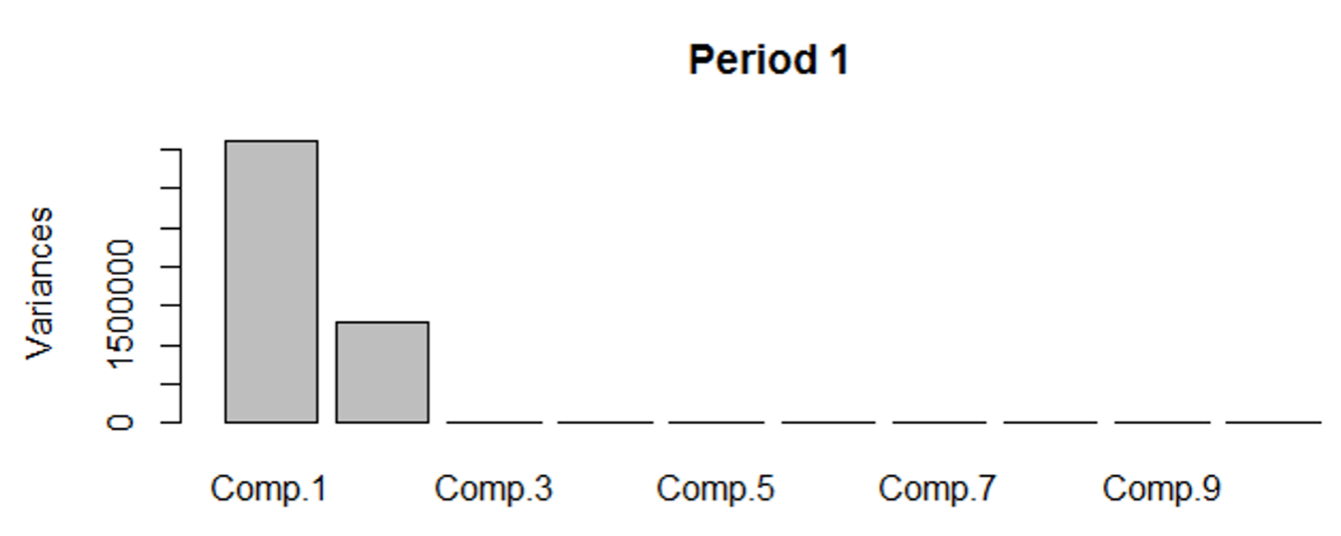
EFA results for period 1.

The EFA method allowed the estimation of the optimal number of factors explaining the greatest part of the variance in the data. As illustrated in Figure 1 for Period 1, the first two factors explained 99.6% of the variance. The first eight factors explained 50.5%, 33.6% and 17.6% of the variance for the Period 2 ~ Period 4 respectively (see Supplementary Fig. 2, Supplementary Fig. 3, Supplementary Fig. 4 respectively Supplementary Material). This indicates that getting closer to the DoD, the variability of prescribed medicines can be explained by a smaller number of factors (clusters), while the prescribed medications appear to cluster. As the patients had the clinically evident dementia, some associated multimorbidity tend to cluster together as well.

**Table 2:** The prevalence of diseases in the Period 1.

Disease Name and Code		Factor 1 diseases prevalence
H06	Acute Bronchitis and Bronchiolitis	4783 (26.65%)
N24	Other soft tissue disorder	4512 (25.14%)
R00	General symptoms	3891 (21.68%)
N05	Osteoarthritis and allied disorders	2899 (16.10%)
N09	Other and unspecified joint disorders	2748 (15.31%)
N14	Other and unspecified back disorders	2607 (14.53%)
K19	Other urethral and urinary tract disorders	2565 (14.29%)
G20	Essential hypertension	2371 (13.21%)
Disease Name and Code		Factor 2 diseases prevalence
H06	Acute Bronchitis and Bronchiolitis	1652 (27.82%)
N24	Other soft tissue disorder	1544 (26.01%)
R00	General symptoms	1209 (22.04%)
N05	Osteoarthritis and allied disorders	976 (16.44%)
N09	Other and unspecified joint disorders	936 (15.77%)
K19	Other urethral and urinary tract disorders	895 (15.07%)
N14	Other and unspecified back disorders	879 (14.80%)
G20	Essential hypertension	793 (13.35%)
Disease Name and Code		Factor 3 diseases prevalence
N24	Other soft tissue disorder	285 (40.59%)
N33	Other bone and cartilage disorders	274 (39.04%)
H06	Acute Bronchitis and Bronchiolitis	274 (39.04%)
N09	Other and unspecified joint disorders	205 (29.21%)
R00	General symptoms	199 (28.37%)
N14	Other and unspecified back disorders	189 (27%)
N05	Osteoarthritis and allied disorders	160 (22.89%)
K19	Other urethral and urinary tract disorders	147 (20.92%)

### Period 1 (0-5 years before DoD)

The 3 factors considering patients with at least three prescribed medicines were identified. Table 1 depicted the demographic information of these three clusters. The cohort of this period comprised of 26,968 patients (35.3% male and 43.34% between 75 and 85 years). Table 2 depicted the prevalence of diseases in this period. The goodness of fit of the model is Root Mean Square Residual (RMRS) 0.01, and the Tucker Lewis Index 93.7. The root mean square error of approximation (RMSEA) resulted in a mediocre fit of the model being 0.07, slightly above the threshold 0.05, a common recommendation of RMSEA value for practice to indicate good fit [36].

#### F1 - Respiratory/urinary infections, arthropathies and rheumatism, and CVD cluster

The first cluster with 66.55% of the patients for this period (17,947 people) accounted for the greatest variance in the data (43.8%). The main classes of medicines included: antibiotics used to treat respiratory and urinary infections, e.g., clarithromycin or erythromycin; analgesics, opioids, non-steroidal anti-inflammatory drugs (NSAIDs), lipid lowering agents, angiotensin enzyme inhibitors, antiplatelet medicines and *gastro-protectants*. These findings were consistent with the diagnostic codes in this cluster: acute bronchitis and bronchiolitis with a prevalence of 63.04%, osteoarthritis and allied disorders with a prevalence of 53.43%, urinary tract disorders with a prevalence of 29.87% and other joint or back disorders, hypertension and dysrhythmias that accounted for a prevalence of 20.66% and 18.02% respectively. The likelihood of medication-disease and medication-medication interactions is high and therefore the presence of other classes of medicines, e.g., eye preparations, can be somewhat explained. Hypertension can be the result of the side effects of NSAIDs, while, hypertension is itself a risk factor for eye conditions [37].

#### F_2_ - Infections, arthropathies and rheumatism, cardio-metabolic and depression cluster

The second cluster represented 22.02% of the cohort (5,938 patients). The identified medicines were almost identical to the cluster *F_1_* with the exception of the medicines used for diabetes, e.g., metformin and gliclazide, hypothyroidism, depression and anxiety. The within-class variation in the prescribed medications was reduced, that is, more patients were taking the same individual medications. The presence of other types of medicines can be explained by the likelihood of drug-drugs or drug-disease interactions. For example, both diabetes and hypertension are risk factors for eye conditions, which had an 18% prevalence in this cluster. Additionally, gastro-protectants can be used to treat or prevent the gastrointestinal side effects of antidepressants/anxiolytics and NSAIDs [32].

#### F_3_ - Arthropathies, rheumatism and osteopathies cluster

The cluster *F_3_* represented 2.6% of patients (701 patients) and identified medicines - alendronic acid, colecalciferol or ergocalciferol and lactulose. The corresponding diseases associated with this cluster were diagnosed with other soft tissue disorder and other bone and cartilage disorders, osteoarthritis, other and unspecified back disorders.

### Period 2 (6-10 years before DoD)

In this period, 4 factors were identified. The total number of the patients in this period was 20,332. 69.7% (14171) of the patients were represented by cluster 1, 3% (610) by cluster 2, 0.3% (61) by cluster 3, and 2.5% (508) by cluster 4 respectively. The goodness of fit of this model was RMRS 0.02, very close to the ideal value of 0. The RMSEA was 0.043 and the *Tucker Lewis index* was 94.1, above the threshold 90.

#### F_1_ - Infections, arthropathies, and CVD cluster

As expected, the first cluster explained much of the variation in the data. It is very heterogeneous in terms of both medicines prescribed and diagnoses. The main categories of medicines were: antibiotics used to treat both urinary and respiratory infections, respectively, such as, cefalexin, trimethropim, oxytetracycline, erythron-mycin, amoxicillin, and cefaclor; analgesics including opioids and NSAIDs; anxiolytics and antidepressants including dosulepin hydrochloride; CVD related medicines; lipid lowering medicines, angiotensin enzyme inhibitors, antiplatelet medicines, and antihypertensives including atenolol, lisinopril and nifedipine; and *gastro-protectants*.

These findings revealed the prevalence of conditions - bronchitis and bronchiolitis (22.3%), soft tissue disorders (20%), osteoarthritis (18%), hypertension (18%) and other urethral and urinary tract disorders (10.1%). Given the high presence of antibiotics for urinary infections and of tamsulosin hydrochloride, different to the first cluster from the Period 1, this cluster was labelled as general infections, and not only acute respiratory infection (ARI).

#### F_2_ - CVD and depression cluster

The cluster F_2_ in this period being almost entirely composed of medicines for CVD, anti-anginal agents, include isosorbide mononitrate, nicorandil, glyceryl trinitrate (all vasodilators used in angina), antihypertensive agents including bisoprolol (beta-blocker), and furosemide (a loop diuretic). Moreover citalopram, an antidepressant, was also present in this cluster. The most prevent diagnoses were angina pectoris (39.2%), and essential hypertension (21%).

#### F_3_ - CMD and arthropathies cluster

The third cluster included antihypertensives (bendroflumethiazide), benzodiazepines for insomnia (nitrazepam), thyroid hormones for hypothyroidism (levothyroxine sodium), and topical anti-inflammatory agents. Of interest, insomnia is often associated with CVD [38]. Acquired hypothyroidism (44.4%), osteoarthritis (34.9%), and essential hypertension (23.8%) were the most prevalent diagnoses in this cluster. It is worth recognising the association between insomnia and CVD.

#### F_4_ - Arthropathies and rheumatism, and CVD cluster

The fourth cluster comprised analgesics, ferrous sulfate, topical antinflammatory preparations and benfro-flumethiazide, with the most prevalent conditions being hypertension (33.6%), and osteoarthritis (36.1%). There are associations between arthritis, hypertension, and other CVD. It is challenging in managing medications to treat patients with both present conditions [39].

### Period 3 (11-15 years before DoD)

In this period, 6 clusters were identified and represented 14,369 patients. Overall, the model fits the data properly with the RMRS 0.02, very close to 0, the RMSEA 0.037 and the Tucker Lewis index 92.1 above 90.0.

#### F_1_ - Infections, arthropathies and CVD cluster

This cluster represents 41.1% (5906) of the patients in this period. Three main categories of medicines were identified: antibiotics for urinary and respiratory infections; antihistamines; corticosteroids for respiratory uses. Expectorants and cough suppressant were common and can be related to the prevalence of bronchitis (20%), urinary tract (10%) disorders and of other respiratory infection (11%). Analgesics and anti-inflammatories, including paracetamol and ibuprofen are also present, as well as antiplatelet and beta-blockers. Interestingly hypertension was common with a prevalence of 15.27%, and based on current guidelines beta-blockers would not be frequently prescribed (NICE guideline [40]). However, beta-blocker management of hypertension was historically common. Other medicines explained the variance of this cluster, such as supplements (iron), and antacids generally prescribed to treat the side effects of the drugs listed above.

#### F_2_ - CVD, ARI, and arthropathies cluster

The second cluster represented 1.25% (180) of the patients, taking medicines, such as antihypertensives (nifedipine and bendroflumethiazide), antibiotics used for respiratory infections and anti-inflammatories. In addition, supplements (iron) are also present. These medicines are consistent with the conditions diagnosed in this cluster: hypertension (28.33%), osteoarthritis and allied disorders (27.7%), and acute bronchitis and bronchiolitis (26.11%). Therefore, this cluster is very similar to the first, except for the infections treated with medicines for the respiratory system.

#### F_3_ - Arthropathies and rheumatism cluster

The third cluster represented 1.16% (167) of the patients with the most common conditions - osteoarthritis and allied disorders (36.5%), and other soft tissue disorders (31%). This is consistent with the types of medications in the cluster, primarily anti-inflammatories including naproxen, diclofenac sodium and rofecoxib. Antibiotics were also present in this cluster. Other medications, such as gastro-protectors was also present and might be prescribed to manage the side effects of some medicines or diseases, like arthritis, which is strongly associated to high blood pressure [41] and may account for the presence of antidiuretics in this cluster.

#### F_4_ - Depression-anxiety cluster

The fourth cluster represented 0.06% (9) of the patients taking opioid analgesics, antidepressants (amitriptyline hydrochloride) and hypnotics(temazepam). Thyroid hormones used to treat hypothyroidism were also present. The association between thyroid function and depression has been identified by a number of studies, namely that individuals with thyroid disorders can be subject to depression and vice-versa [42].

#### F_5_ - CMD cluster

The fifth cluster represented 1.4% (201) of the patients and was very homogeneous. The most common medicines included simvastatin, amlodipine, furosemide and isosorbide mononitrate for angina pectoris and hypertension, and metformin hydrochloride for diabetes. This can be reflected by the prevalence of angina pectoris (42%), diabetes mellitus (41%), disorders of lipoid metabolism (25%) and essential hypertension (22%).

#### F_6_ - Dermatologic disease cluster

The sixth cluster represented 0.9% (129) of the patients taking topical corticosteroids, non-surgical adhesive tapes and gastrointestinal agents and bulk forming drugs.

### Period 4 (16 - 20 years before DoD)

The Period 4 covered 7,660 patients with 146 different prescribed medicines. Five clusters were identified with the patients taking at least three medicines. The most populated was the first cluster with 5.5% of the patients (421), followed by the second with 2.4% (184), the third with 2.1% (161), whilst clusters 4 and 5 were less populated representing 0.3% (23) and 0.05% (4) of the patients respectively. The very small number of patients covered in the clusters 4 and 5 would make drawing any inference moot, so two clusters will not be described any further.

The goodness of fit of this model was very good in terms of both RMRS and RMSEA, being 0.03 (close to 0) and 0.033 (less than the 0.05) respectively. But the model was poor in terms of Tucker Lewis index 59.3 (under the threshold 90).

#### F_1_ -Infections, arthropathies, and cardio-vascular cluster

The first cluster included antibiotics used to treat urinary and respiratory infections and supplements (iron). A smaller range of medicines used to treat hypertension were identified, together with antacids. Supporting these findings, the most prevalent conditions were acute bronchitis and bronchiolitis (29.4%), other soft tissue disorders, other and unspecified joint or back disorders, and osteoarthritis range between approximately 20% and 22%. The prevalence of essential hypertension is 16.7%.

**Figure 2.**
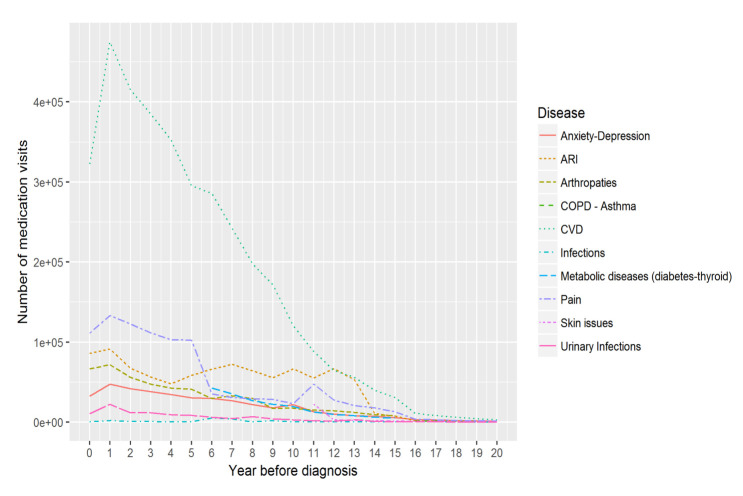
Yearly number of medication visits for each disease in all periods before the diagnosis.

#### F_2_ - Depression-anxiety, and ARI cluster

The two main categories of medicines prescribed were analgesics including codeine phosphate and paracetamol: antidepressants and tranquillizers including temazepam and amitriptyline hydrochloride. Gastro protectors and laxatives were also present which may be explained by the possible side effects of antidepressants or opioids[32]. The most prevalent condition was acute bronchitis and bronchiolitis (21.62%).

#### F_3_ - ARI and CVD cluster

The third cluster is quite homogeneous in terms of medicine composition, with the presence of medicines used to treat asthma and respiratory infections including beclometasone dipropionate and erythromycin respectively, and cardiovascular conditions including diltiazem hydrochloride and diuretics. Medications used to treat stomach and oesophagus problems were also present. These observations were consistent with the prevalence of asthma (40%), acute bronchitis and bronchiolitis (34.3%) and other acute respiratory infections (18%), angina (16%) and hypertension (14%).

**Figure 3.**
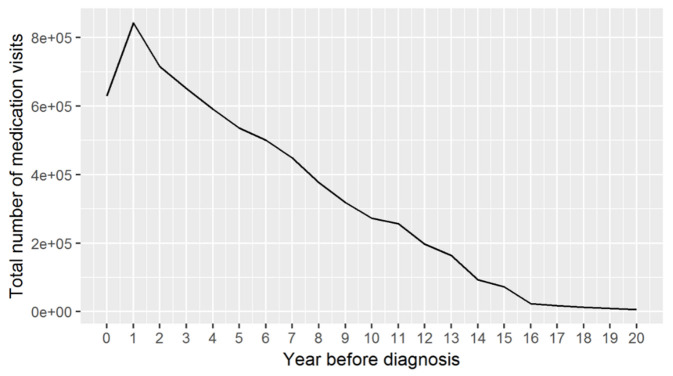
Yearly total number of medications in all periods before the diagnosis.

## DISCUSSIONS

This study demonstrated that patterns of polypharmacy varied significantly as well as the developmental patterns of dementia diagnosis, although an assessment of causality has not been possible[6]. Understanding these patterns would contribute to assessing appropriateness of prescribing medications prior to DoD, provide opportunities for early intervention in prescribing, and support the identification of medications at risk of drug-drug interactions. Previous studies assessing medication appropriateness in the elderly, including the development and use of decision support tools, have not focused specifically on PwD [24]. This study provides a starting point to address the under-researched issues of variations of polypharmacy among PwD before the DoD, and their relationships with multiple morbidities.

Most studies on polypharmacy predominantly considered the influence of comorbidity and polypharmacy on the prescription of particular types of medication [43, 44, 45] or on the outcome of the disease being treated [2, 46, 47]. Swanson and Carnahan noted that vascular diseases, including hypertension and atrial fibrillation are common in PwD [48]. Poblador-Plou et al. found that cardiac arrhythmia, skin ulcers, thyroid disorders, insomnia, anxiety, and congestive heart failure are highly prevalent in both men and women with dementia[49]. These diseases were also highly prevalent in the patterns identified by our study. Moreover, as shown in our study, the Period 1 recognised an association between arthritis and dementia, but the nature of the relationship is unclear [50]. Consideration of confounding factors for any relationship is often required. For example, managing arthritis in patients with asthma requires consideration of medication choice as a proportion of patients are unable to manage NSAIDs which can cause asthma exacerbation [51]. NSAIDs increase the risk of developing hypertension and CVD, the recognised risk factors for dementia [50]. Moreover, the relationship between asthma and pulmonary diseases with dementia has been noted [52]. Specifically, COPD patients with frequent chest or respiratory infections, are estimated to have a 79% higher risk of developing dementia compared with patients without [52]. The first two clusters identified in the Period 1 by our study included these same conditions (comorbidities) and could be considered to validate the relationship between these comorbidities and dementia.

**Figure 4.**
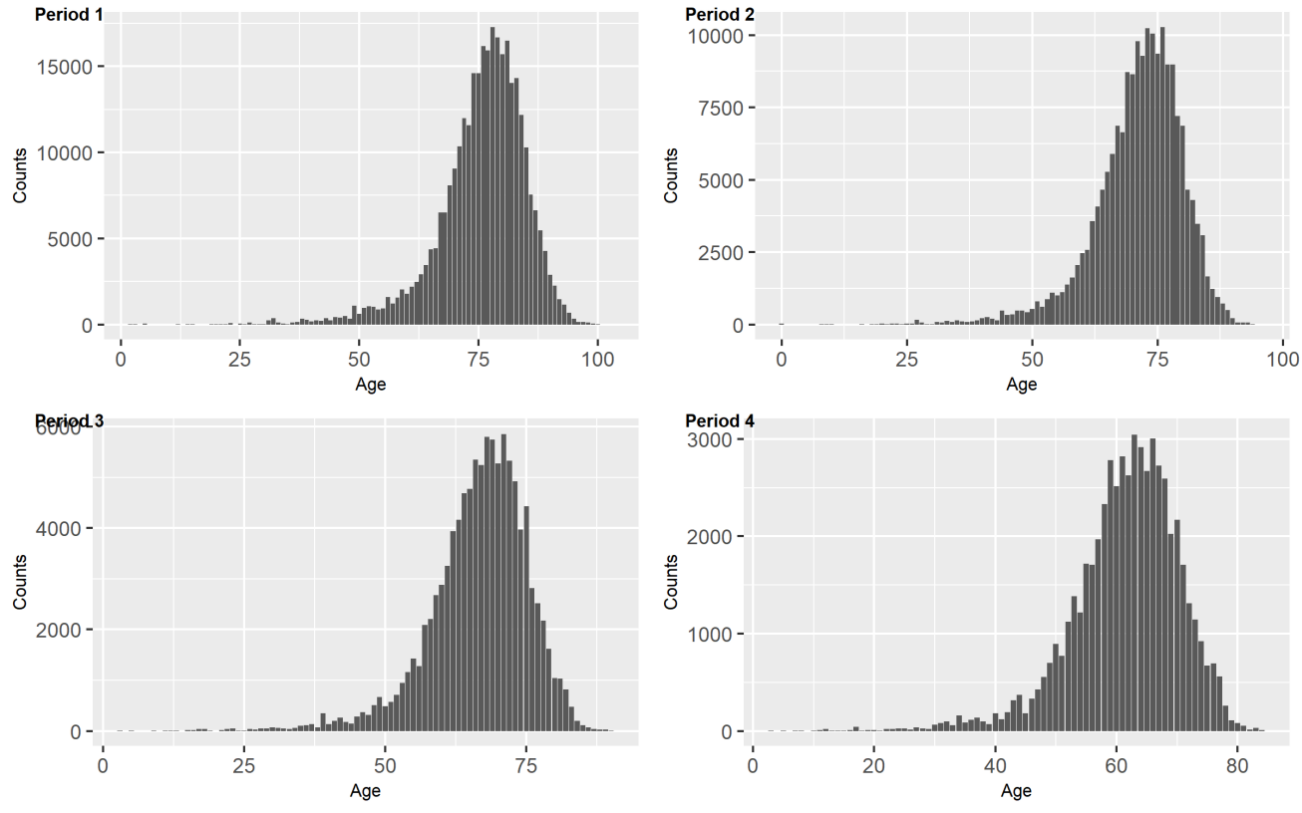
Variations of number of diseases by age for each period.

Our study considered variations in polypharmacy patterns over a 20 year long period prior to DoD. The closer the patients approached the date of DoD, the fewer the patterns were generated whilst the number of medicines included in the first cluster increased, considering the first cluster explained the maximal variance of the data. This suggests that the closer the patients approached the date of DoD, the greater the number of comorbidities in patients became and the greater the proportion of the population represented in the first cluster. In other words, more patients shared the homogeneous comorbidities and associated medicines as they were closer to the DoD.

As the number of medicines increase the likelihood of multiple drug-drug interactions also increases, with a corresponding increased risk of developing cognitive and functional decline [53]. This observation necessitates future longitudinal studies to confirm any potential causal association resulting from the patterns of polypharmacy discovered. It also provides an impetus for tackling the issue of medicine interactions in patients with comorbidities while taking multiple medicines at risk of developing dementia.

Our study revealed that the annual number of medication related GP visits increased along with approaching the DoD and was consistent across all diseases (Fig. 2 and Fig. 3). In particular, CVD related medication visits increased dramatically from 12 years before the diagnosis to the year before the DoD. This is consistent with one existing study suggesting that the number of prescribed medicines increases along with patients aging [54]. In the Period 1, covering the five years before the diagnosis, pain related medication visits became the second most important element after CVD ones, overtaking the ARI and arthropathy related visits, which slowly decreased between year 4 and 7 before the diagnosis. Two interesting observations are noteworthy:

1) The medication visits related to chronic diseases, including CVD, anxiety, and depression, and arthropathies, increased throughout the 20 years before the diagnosis, while the non-chronic disease related visits including infections changed irregularly throughout the same period, except the Period 1 showing a noticeable increase in all of these visits.
2) In the year of the DoD, the number of medication visits dropped for all the disease categories. The reasons for this could be complex. Such a reduction in prescribing visits may reflect changes where dementia is suspected, including, but not limited to, increased hospitalisation, and avoidance of GP[55]. It could also be explained by early adoption of national guidelines to avoid inappropriate polypharmacy in elderly patients [56] once the dementia was suspected. The total number of medications for chronic diseases and, specifically, cardiovascular medications actually declined more in patients recently diagnosed with dementia.

As shown in Figure 4, another interesting observation is that numbers of diseases by age before dementia diagnosis match the revealed pattern in each period, from total 315,358 diseases in period 1, total 187,423 diseases in period 2, total 106,697 diseases in period 3, to total 57,286 diseases in period 4.

Our study using the large dataset with over 33,000 PwD generated the new and reliable results, confirmed by the goodness of fit related indexes. However, this study has a few limitations. Firstly, as this is a dementia cohort study, there is no control cohort. In other words, this study cannot provide an insight into how patterns of polypharmacy change between dementia patients and non-dementia participants, and how aging factor can contribute to such variations. This topic merits further research in the future. Secondly, there is often a long lead time until dementia is formally diagnosed in individuals. This could affect the way in which medications may be clustered temporally. Thirdly, the GP dataset collected does not provide details of prescribed medicines on doses, so it is not possible to generate clusters controlled for dose levels, which is an important consideration when assessing appropriate prescribing[57]. Fourthly, our analysis did not differentiate between different types of dementia, e.g., Alzheimer's disease or Lewy Body dementia. The stratification of the PwD could provide insights into clusters of medicines and comorbidities associated with a particular dementia type.

### Conclusions

The patterns of polypharmacy varied significantly along with patients developing towards dementia. As the development towards dementia, the associative diseases tended to cluster with a larger prevalence in each cluster. As farther away towards the DoD, the clusters of polypharmacy tended to be clearly distinct between each other, resulting in an increasing number of patterns, but each identified pattern has a smaller prevalence. These varying patterns would inform “safe- prescribing” practice before dementia diagnosis in terms of selecting low anticholinergic burden medicines to minimise their impact on cognitive impairments. The drug-drug interactions as predictors of DoD to support the practice of personalised care merits further research.

## Supplementary Materials

The Supplementary data can be found online at: www.aginganddisease.org/EN/10.14336/AD.2021.0829.
