# Supplementary material for: Identifying Dynamic Patterns of Polypharmacy for Patients with Dementia from Primary Care Electronic Health Records: A Machine Learning Driven Longitudinal Study
Source: Aging Dis. 2023 Apr 1;14(2):548–59. doi: 10.14336/AD.2022.0829 (PMC10017143; doi:10.14336/AD.2022.0829)
Supplement: Supplementary file 1 — The Supplementary data can be found online at: www.aginganddisease.org/EN/10.14336/AD.2021.0829. [file AD-14-2-548-s.pdf]

SUPPLEMENTARY DATA

Identifying Dynamic Patterns of Polypharmacy for Patients with Dementia from Primary Care Electronic Health Records: A Machine Learning Driven Longitudinal Study

Elisabetta Longo[1], Bruce Burnett[2], Sarah Bauermeister[3], Shang-Ming Zhou[4*]

© 2022. Longo E et al. Published online at http://www.aginganddisease.org/EN/10.14336/AD.2022.0829

SUPPLEMENTARY DATA

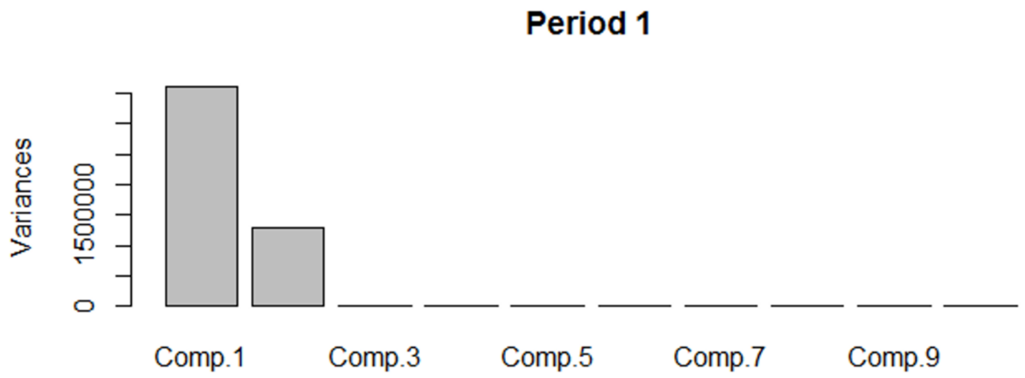

Supplementary Figure 1. PCA results for period 1.

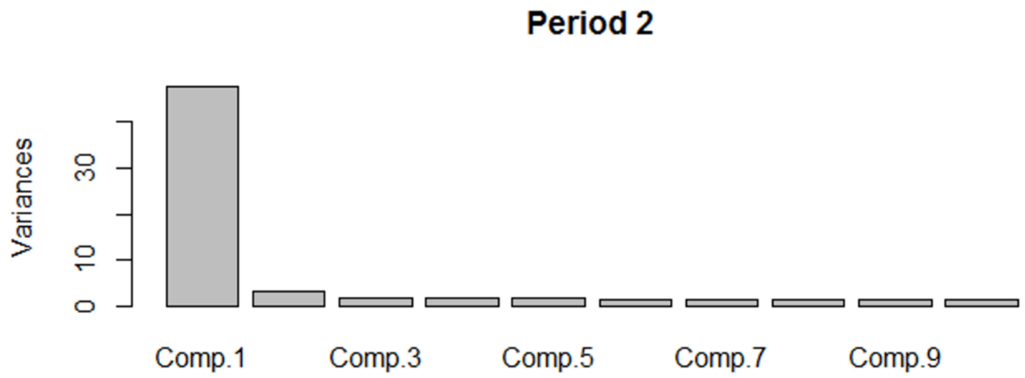

Supplementary Figure 2. PCA results for period 2.

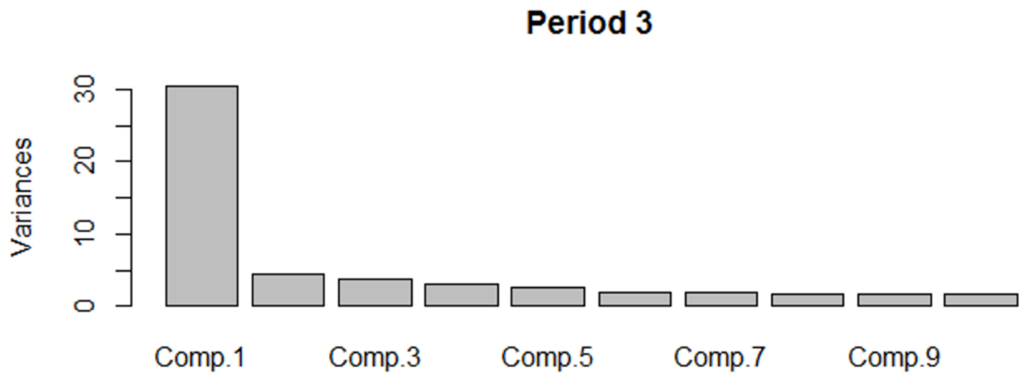

Supplementary Figure 3. PCA results for period 3.

© 2022. Longo E et al. Published online at http://www.aginganddisease.org/EN/10.14336/AD.2022.0829

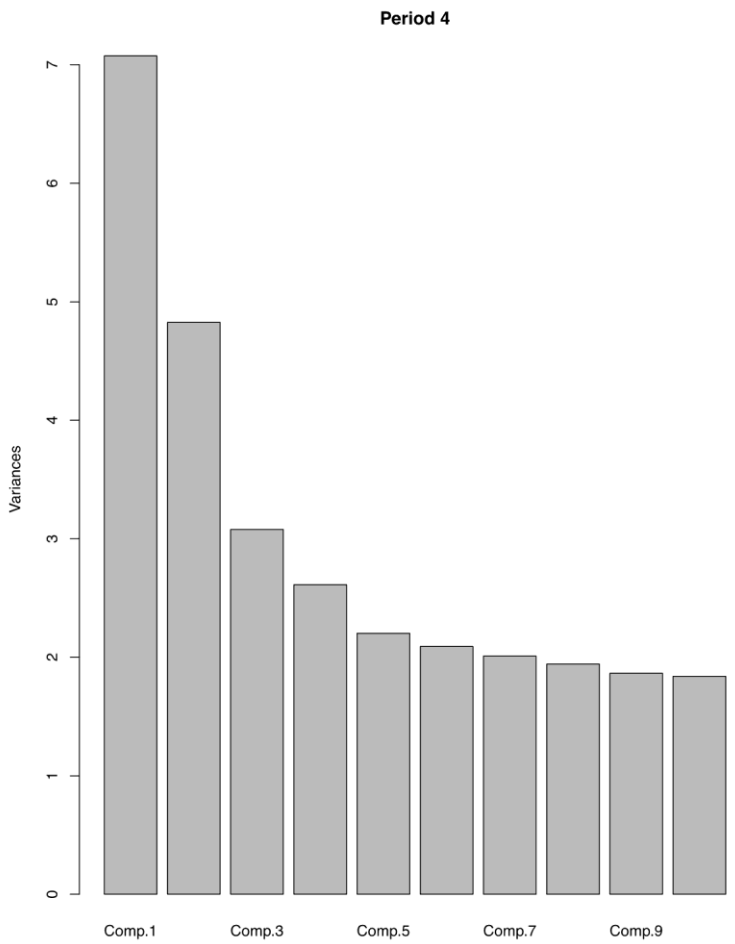

Supplementary Figure 4. PCA results for period 4.

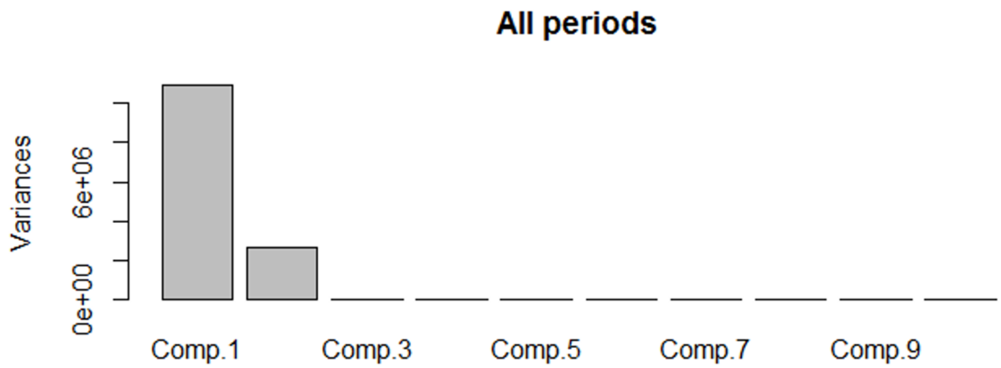

Supplementary Figure 5. PCA results for all periods before the diagnosis.

© 2022. Longo E et al. Published online at http://www.aginganddisease.org/EN/10.14336/AD.2022.0829

SUPPLEMENTARY DATA

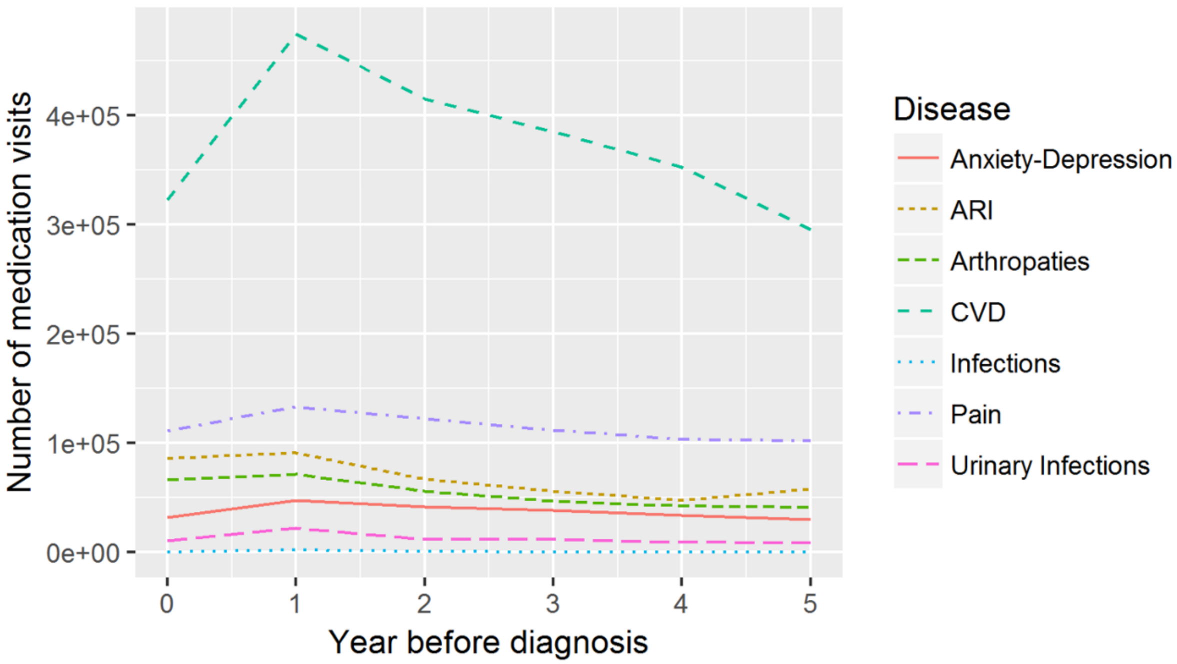

Supplementary Figure 6. Yearly number of medication visits for each disease in Period 1.

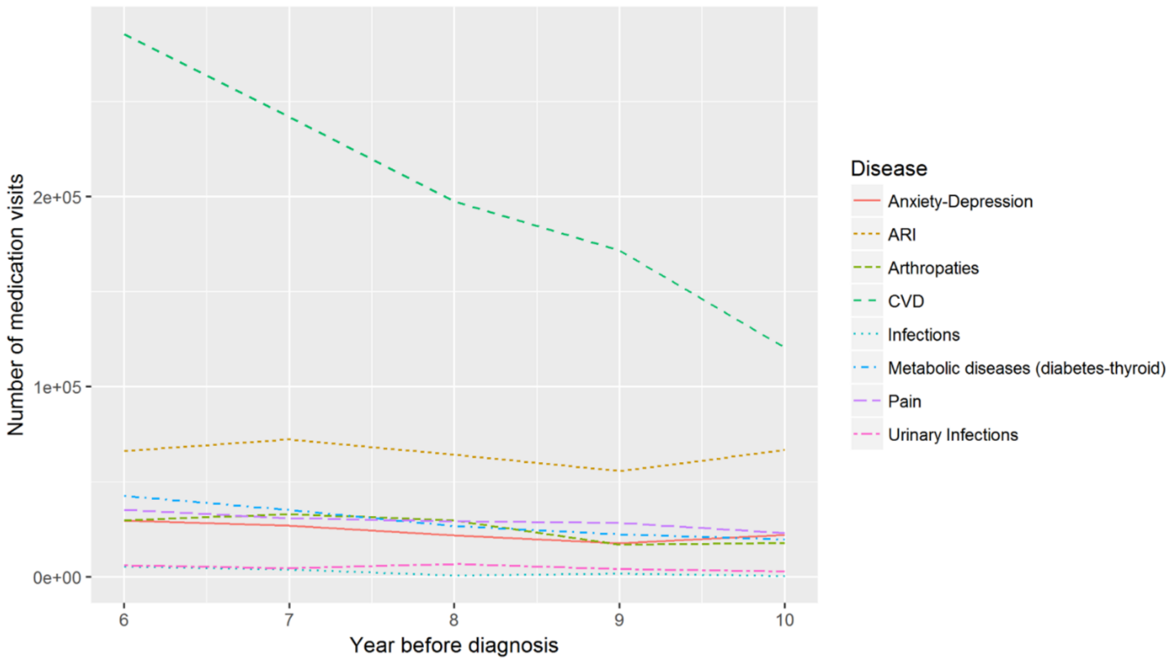

Supplementary Figure 7. Yearly number of medication visits for each disease in Period 2.

© 2022. Longo E et al. Published online at http://www.aginganddisease.org/EN/10.14336/AD.2022.0829

SUPPLEMENTARY DATA

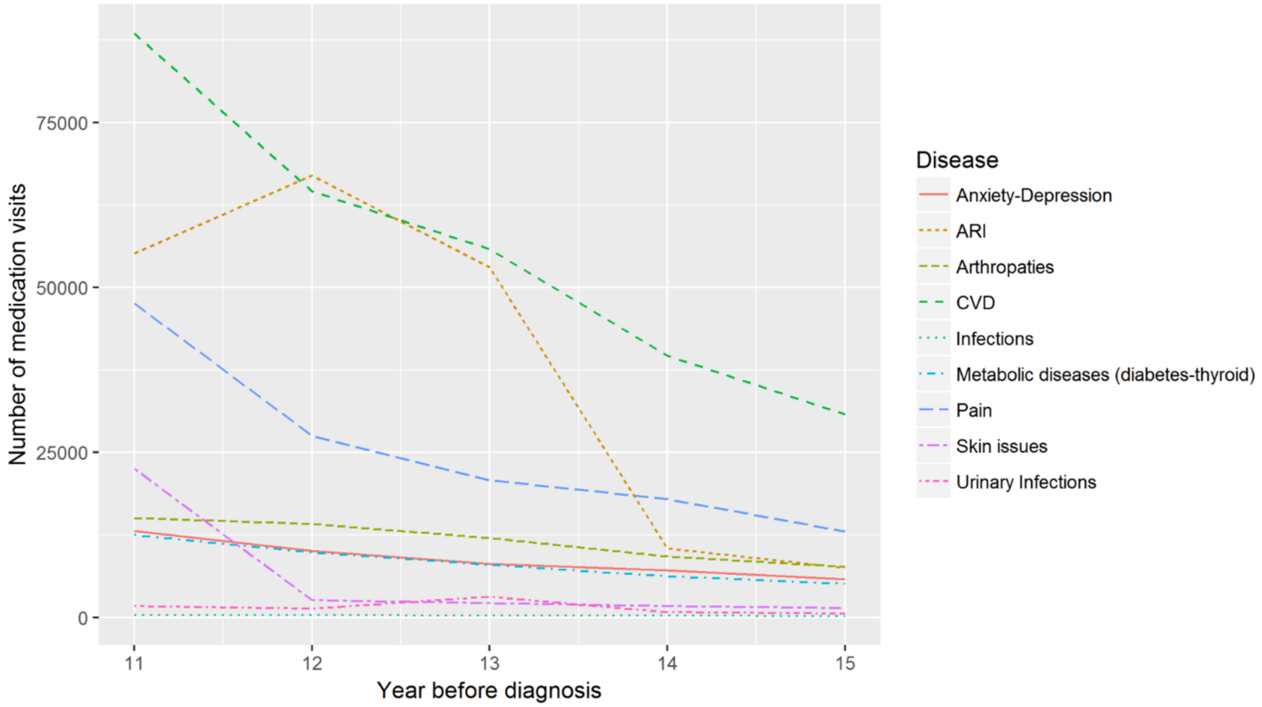

Supplementary Figure 8. Yearly number of medication visits for each disease in Period 3.

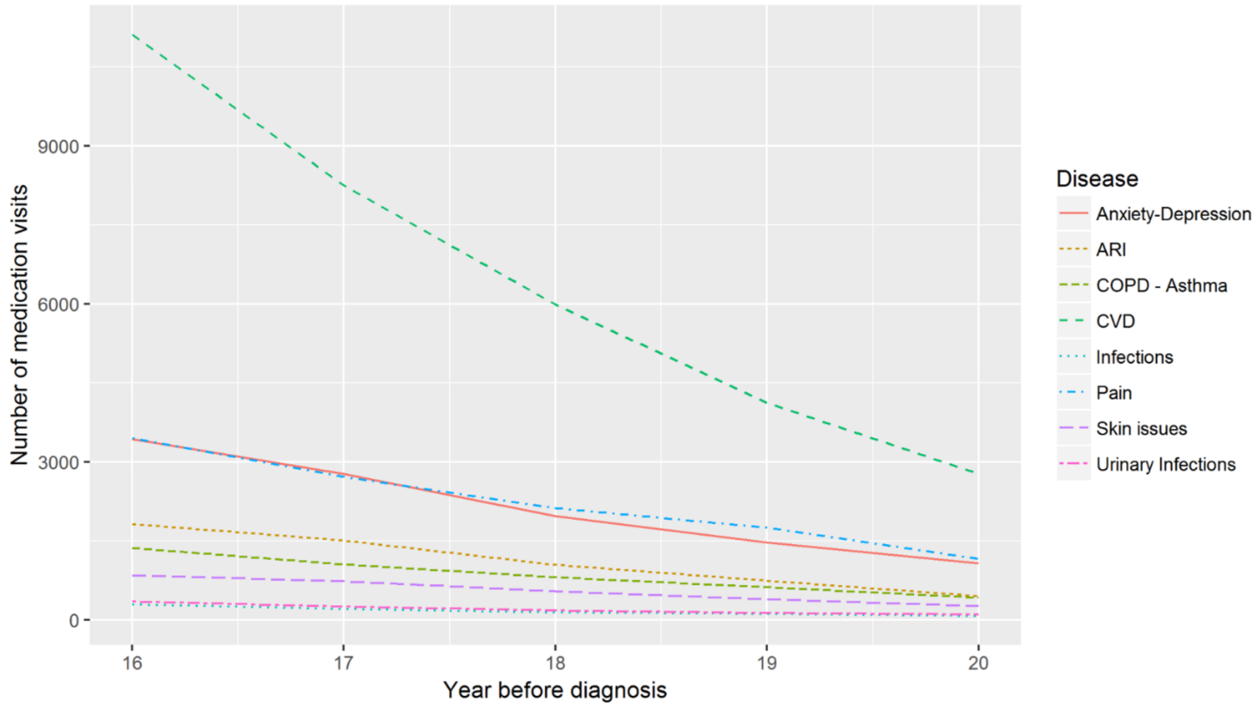

© 2022. Longo E et al. Published online at http://www.aginganddisease.org/EN/10.14336/AD.2022.0829

SUPPLEMENTARY DATA

Supplementary Figure 9. Yearly number of medication visits for each disease in Period 3.

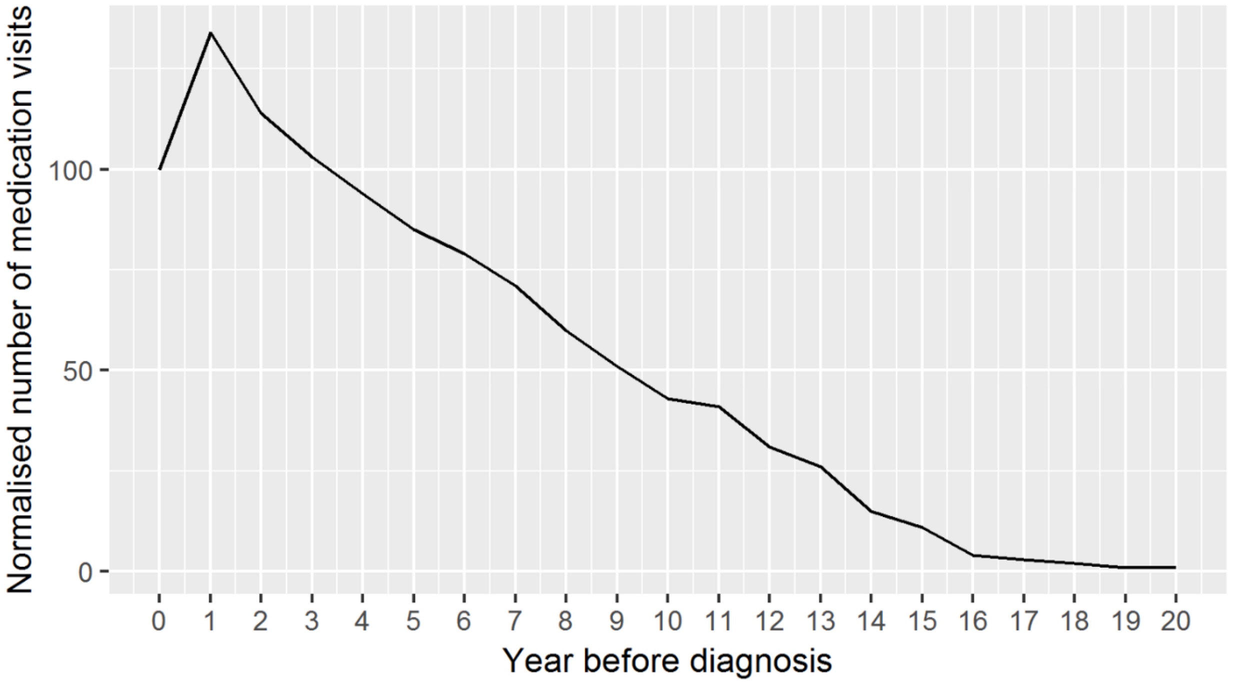

Supplementary Figure 10. Yearly normalized total number of medication in all periods before the diagnosis.

Supplementary Table 1. Factor loadings for period 1 (loadings > |0.3|)

	MR1	MR2	MR4	MR3
**a23**	0.955288878			
**a62**		0.99031155		
**a6b**		0.969997076		
**a6c**		0.987080422		
**a83**	0.7672693			
**ab2**	0.992568512			
**ac7**	0.35063882			
**ae1**	0.457969207			0.378429356
**ae4**	0.911042544			
**b21**		0.996335297		
**b31**		0.940093174		
**b51**		0.996213749		
**bd3**	0.973053101			
**bdf**	0.317723417			
**bi3**	0.997477594			
**bi5**		0.99848576		
**bi6**	0.980359604			
**bl3**		0.974722315		
**bl8**	0.96996276			
**blb**	0.980715776			
**blz**	0.743704013	0.657505516		
**bs1**	0.604968614	0.792865971		
**bu2**	0.922565587	0.383184828		
**bu5**	0.992205901			

© 2022. Longo E et al. Published online at http://www.aginganddisease.org/EN/10.14336/AD.2022.0829

**bxd**	0.959233682			
**bxi**	0.754053965	0.531184407		
**c13**	0.746170409	0.663440366		
**d1a**		0.99789586		
**d1d**	0.979965648			
**d21**	0.672703875			
**d71**	0.983957308			
**da4**	0.900879364	0.411448435		
**da9**		0.840245242		
**di2**	0.938464732	0.341004068		
**dia**	0.961026388			
**dib**		0.985833487		
**dji**	0.817028476			
**e15**	0.786856022	0.615075044		
**e22**	0.402167979	0.912534965		
**e31**	0.927247882	0.371308098		
**e61**	0.992462198			
**e69**		0.994114859		
**e91**	0.997111622			
**e92**	0.991716585			
**ecc**		0.992386106		
**eg1**	0.971082384			
**eg6**		0.960785511		
**ej7**	0.975219734			
**f35**		0.839989986		
**f41**		0.969389979		
**f92**		0.944412448		
**fe6**		0.983732286		
**fo4**				0.625276471
**i15**	0.806448566			
**i31**	0.996003953			
**i33**		0.9003218		
**ip3**				0.815615915
**j22**	0.938533425	0.331025254		
**j28**	0.544581804	0.837467011		
**j2c**		0.991519627		
**ja1**	0.996961657			
**k3g**	0.992084917			
**ka2**	0.744543488			
**l71**	0.997448006			
**m11**		0.982497397		
**m21**		0.992016227		
**m41**	0.997940918			
**m45**	0.891879269			
**m48**	0.405542502		0.745627902	
**m49**	0.997551475			
**m4b**			0.891025711	
**md2**	0.988350357			
**mif**		0.993349835		
**n47**	0.716376159	0.694586796		
**n4b**	0.402224064	0.913712138		
**p16**	0.997966861			
**p1a**	0.954272213			
**p1n**	0.559502389	0.827080678		
**p1w**	0.997966241			
**p5j**	0.996550372			
**ps7**		0.996885925		
**puh**	0.458275032	0.881706972		

© 2022. Longo E et al. Published online at http://www.aginganddisease.org/EN/10.14336/AD.2022.0829

Supplementary Table 2. Factor loadings for period 2 (loadings > |0.3|).

	MR1	MR2	MR3	MR4	MR5
a23	0.991563065				
a4e	0.999246318				
a6b	0.993674132				
a6c	0.852814102				
ab2	0.995226178				
ac7	0.705900266				
aj1	0.936846505				
b21				0.40955365	
b31		0.545134834			
b51					0.652532477
bd3	0.973158456				
bde	0.879422221				
bdf		0.393913412			
bi3	0.998277784				
bi5			0.984862208		
bi6	0.876392103				
bl3		0.521079883			
bl5			0.997424645		
bl8	0.960073168				
blf		0.385485784			
blz		0.31580185			
bu2	0.999132518				
bu5	0.704525197				
bxd	0.95011798				
bxi	0.986421609				
c13	0.999253546				
c1D	0.995131886				
c61	0.999000676				
c8o	0.999129868				
cg7	0.987910469				
ch1	0.995292868				
d18				0.564996589	
d1a					0.801009972
d1d	0.682368112				
d21	0.411112422				
d75	0.991566501				
da9		0.307369606			
di2	0.999368578				
dia	0.817358697				
e15	0.999239034				
e31	0.999368464				
e61	0.936796524				
e69	0.998245195				
e78	0.998264451				
e91	0.997872553				
ecc	0.997056566				
f41	0.65364633				
f92				0.421247718	
fe6	0.98808538				
gc7	0.840496776				
i31	0.998085163				
j22	0.996519342				
j28	0.922546763				

© 2022. Longo E et al. Published online at http://www.aginganddisease.org/EN/10.14336/AD.2022.0829

**ja1**	0.994831935				
**ja3**				0.540705928	
**ka2**	0.915325823				
**ka8**	0.983784679				
**l81**	0.957684257				
**m11**	0.993321178				
**m21**	0.968426674				
**m48**	0.999011955				
**n47**	0.993074497				
**n4b**	0.98320194				
**p1a**	0.998829821				
**p5j**	0.997245813				
**ph7**	0.995364809				
**puh**	0.994521981				

Supplementary Table 3. Factor loadings period 3 (loadings > |0.3|)

	**MR1**	**MR2**	**MR3**	**MR4**	**MR6**	**MR5**
**a23**	0.97774215					
**a62**			0.713375799			
**a69**				0.837836079		
**ab2**					0.346533968	
**b21**		0.834568593				
**b31**			0.333707126			0.315092225
**bd3**	0.956592032					
**bde**	0.963929742					
**bi6**	0.981332197					
**bl3**						0.446914743
**bl8**		0.851822708				
**blb**						0.306438207
**bu2**	0.896238843					
**bxd**						0.378597541
**c13**	0.99940175					
**c61**	0.998338033					
**c84**	0.999569935					
**c8o**	0.99953377					
**cg7**	0.997145285					
**ch1**	0.99208844					
**d1a**				0.542926583		
**d21**	0.747573774					
**d71**				0.81241472		
**di2**	0.998763593					
**dia**	0.919914846					
**dj4**				0.3009861		
**dj8**				0.398009574		
**e15**	0.995450158					
**e31**	0.999639776					
**e61**	0.99586688					
**e69**	0.996965189					
**e91**	0.998545663					
**e92**		0.927762751				
**ecc**	0.99739151					
**ef1**			0.8320916			
**f41**						0.312328227
**f92**				0.39099617		
**fh1**	0.999555313					
**i15**		0.959993238				

© 2022. Longo E et al. Published online at http://www.aginganddisease.org/EN/10.14336/AD.2022.0829

**i31**	0.970125409					
**j22**			0.311349146			
**j28**	0.851763614					
**j2c**			0.900241775			
**j43**	0.998691014					
**ja1**		0.926258733				
**jA1**			0.912976511			
**m21**	0.988788906					
**m41**					0.301955071	
**m48**	0.99950287					
**n4b**	0.999364547					
**n4g**	0.99954467					
**n4m**	0.999572921					
**p16**	0.999036981					
**p1a**	0.99907234					
**p5j**					0.321069784	
**puh**	0.878128916					

Supplementary Table 4. Factor loadings for period 4 (loadings > |0.3|)

	**MR1**	**MR2**	**MR3**	**MR4**	**MR5**
**a23**	0.69964114				
**a69**		0.800845128			
**a6b**			0.300055792		
**a83**					0.638101021
**ab2**		0.406457922		0.330888302	
**ae1**		0.494533524			
**b21**	0.905282996				
**bd3**		0.316137857			
**bl5**			0.317644432		
**bl8**	0.726827894				
**cg1**		0.429261683			
**d1a**		0.616525764			
**d1d**				0.409319353	
**d71**		0.601216065			
**di2**		0.636052045			
**dj4**		0.895819407			
**e22**	0.586173023				
**e31**	0.948121049				
**e75**	0.542449593				
**e92**	0.953898709				
**ecc**	0.382153089				
**eg6**	0.353101174				
**f92**		0.323888971			
**fe6**					0.30871439
**i15**	0.983058009				
**j2a**	0.956258161				
**ka2**				0.338922906	
**m11**				0.493089846	
**m25**				0.643204095	
**m4b**				0.4526365	
**me4**				0.62676288	
**n47**			0.317865161		
**p43**			0.369071193		
**p5j**					0.891589614

© 2022. Longo E et al. Published online at http://www.aginganddisease.org/EN/10.14336/AD.2022.0829

SUPPLEMENTARY DATA

Supplementary Table 5. Factor loadings for all periods (loadings > |0.3|).

	MR1	MR2	MR3
**a12**	0.876554832		
**a23**	0.982827437		
**a4e**	0.979104935		
**a62**		0.968653533	
**a69**			
**a6b**	0.509655782	0.854692016	
**a6c**		0.967908853	
**a6h**		0.966590094	
**a83**	0.620142373		
**aa6**	0.995365735		
**ab2**	0.993505854		
**ac7**	0.540835178		
**ae1**	0.324056858		0.434093735
**ae4**	0.872203932		
**aj1**	0.874675005		
**b21**		0.987379246	
**b31**		0.882362595	
**b51**		0.987585972	
**bd3**	0.976640429		
**bde**	0.855126178		
**bi3**	0.998833496		
**bi4**	0.998831438		
**bi5**		0.999402358	
**bi6**	0.979041904		
**bk3**	0.389483507		
**bk5**	0.974094737		
**bl3**		0.929441413	
**bl5**		0.999400772	
**bl8**	0.934420672		
**blb**	0.959761536		
**ble**		0.99774712	
**blz**	0.749897951	0.623789551	
**bs1**	0.62686999	0.772146667	
**bu1**	0.930090015		
**bu2**	0.96910818		
**bu5**	0.988791452		
**bxd**	0.967028519		
**bxg**	0.830602718		
**bxi**	0.935668612		
**bxk**	0.775494317		
**c11**	0.900590754		
**c13**	0.959284589		
**c14**	0.980418151		
**c1D**	0.99573571		
**c31**	0.790783586		
**c43**	0.781122834		
**c61**	0.902634837	0.428442979	
**c66**	0.995626753		
**c67**	0.996574483		
**c84**	0.999263545		
**c8n**	0.990384035		
**c8o**	0.999252335		
**cg7**	0.984032231		
**ch1**	0.998662233		
**d1a**		0.993427836	

© 2022. Longo E et al. Published online at http://www.aginganddisease.org/EN/10.14336/AD.2022.0829

**d1d**	0.970927119		
**d21**	0.666143816		
**d61**	0.823127243		
**d71**	0.955047391		
**d75**	0.931257626		
**d79**		0.998576052	
**da4**	0.902891088	0.380250263	
**da9**		0.771400818	
**di1**	0.998350893		
**di2**	0.982784454		
**dia**	0.943655932		
**dib**		0.961480373	
**dic**	0.872774479		
**dj1**	0.846608929	0.529329292	
**dj4**	0.654333491		
**dji**	0.698361732		
**djk**	0.998751833		
**dn3**	0.918180101		
**dnb**		0.994746523	
**dnj**	0.566028938		
**dqA**	0.988117681		
**e15**	0.916503798	0.398576179	
**e22**	0.429186647	0.897984285	
**e31**	0.984322327		
**e3z**	0.899790768	0.435135753	
**e61**	0.991030636		
**e69**	0.513291778	0.85502827	
**e75**	0.992706962		
**e78**	0.998397953		
**e91**	0.999113836		
**e92**	0.970941957		
**ecc**	0.308071986	0.950626446	
**ef1**	0.99612203		
**eg1**	0.955688614		
**eg6**		0.951723148	
**ei1**	0.986638326		
**ej7**	0.938718773		
**f33**	0.998680095		
**f35**		0.705642851	
**f41**		0.927011107	
**f92**		0.862945977	
**fe6**	0.427359225	0.895116537	
**fh1**	0.999436632		
g42	0.984456967		
gc7	0.629589228		
gh5	0.998103251		
h34	0.999288385		
**i15**	0.580789238		
**i31**	0.997444571		
**i33**		0.853325416	
**ip3**			0.446171438
**iz1**			0.431500568
**j11**	0.997970219		
**j22**	0.98134675		
**j28**	0.61193497	0.789492663	
**j29**	0.956176449		
**j2c**		0.955238271	
**j43**	0.998660442		
**j61**		0.944716458	

© 2022. Longo E et al. Published online at http://www.aginganddisease.org/EN/10.14336/AD.2022.0829

**ja1**	0.995815573		
**jA1**			0.396430252
**k3g**	0.975820316		
**k69**	0.994435707		
**k83**	0.824015033		
**k85**		0.999131005	
**k8f**	0.654120007		
**k8k**	0.941156008		
**k8l**	0.919402049		
**ka2**	0.817457555		
**ka4**	0.874898307		
**ka8**		0.996277705	
**l35**	0.999466048		
**l71**	0.998000096		
**l81**	0.850404878		
**le2**	0.867669896		
**lf1**	0.914317156	0.397531085	
**m11**	0.380310674	0.923017742	
**m12**	0.90137918		
**m21**		0.955619111	
**m22**	0.986499581		
**m25**		0.974937065	
**m27**		0.992694534	
**m41**	0.999282069		
**m45**	0.74236058		
**m48**	0.999065311		
**m49**	0.998161335		
**mc5**	0.998184128		
**md2**	0.980386933		
**me4**	0.999206096		
**mif**		0.992032772	
**n47**	0.781461858	0.621846902	
**n4b**	0.661069084	0.748833751	
**n4g**	0.999437193		
o42	0.951405897		
oaz	0.99346923		
**p16**	0.999470237		
**p1a**	0.997782111		
**p1i**	0.995168342		
**p1m**	0.998214712		
**p1n**	0.885387201	0.463035625	
**p1u**			0.476934285
**p1w**	0.999383158		
**p59**	0.999043568		
**p5j**	0.99749853		
**pA2**			0.463239793
**ph7**	0.994042534		
**ps7**		0.996894852	
**puh**	0.838631248	0.525133591	

Supplementary Table 6. Diseases prevalence in period 1.

**Disease Name and Code**		**Factor 1 diseases prevalence**
H06	Acute Bronchitis and Bronchiolitis	4783 (26.65%)
N24	Other soft tissue disorder	4512 (25.14%)
R00	General symptoms	3891 (21.68%)
N05	Osteoarthritis and allied disorders	2899 (16.10%)
N09	Other and unspecified joint disorders	2748 (15.31%)
N14	Other and unspecified back disorders	2607 (14.53%)

© 2022. Longo E et al. Published online at http://www.aginganddisease.org/EN/10.14336/AD.2022.0829

K19	Other urethral and urinary tract disorders	2565 (14.29%)
G20	Essential hypertension	2371 (13.21%)
**Disease Name and Code**		**Factor 2 diseases prevalence**
H06	Acute Bronchitis and Bronchiolitis	1652 (27.82%)
N24	Other soft tissue disorder	1544 (26.01%)
R00	General symptoms	1209 (22.04%)
N05	Osteoarthritis and allied disorders	976 (16.44%)
N09	Other and unspecified joint disorders	936 (15.77%)
K19	Other urethral and urinary tract disorders	895 (15.07%)
N14	Other and unspecified back disorders	879 (14.80%)
G20	Essential hypertension	793 (13.35%)
**Disease Name and Code**		**Factor 3 diseases prevalence**
N24	Other soft tissue disorder	285 (40.59%)
N33	Other bone and cartilage disorders	274 (39.04%)
H06	Acute Bronchitis and Bronchiolitis	274 (39.04%)
N09	Other and unspecified joint disorders	205 (29.21%)
R00	General symptoms	199 (28.37%)
N14	Other and unspecified back disorders	189 (27%)
N05	Osteoarthritis and allied disorders	160 (22.89%)
K19	Other urethral and urinary tract disorders	147 (20.92%)

Supplementary Table 7. Diseases prevalence period 2.

**Disease Name and Code**		**Factor 1 diseases prevalence**
H06	Acute Bronchitis and Bronchiolitis	3154 (22.26%)
N24	Other soft tissue disorder	2877 (20.30%)
N05	Osteoarthritis and allied disorders	2561 (18.07%)
G20	Essential hypertension	2559 (18.06%)
N09	Other and unspecified joint disorders	1983 (13.99%)
N14	Other and unspecified back disorders	1981 (13.98%)
R00	General symptoms	1863 (13.15%)
H05	Other acute upper respiratory infections	1551 (10.95%)
**Disease Name and Code**		**Factor 2 diseases prevalence**
G33	Angina pectoris	239 (39.20%)
H06	Acute Bronchitis and Bronchiolitis	204 (33.44%)
N24	Other soft tissue disorder	167 (27.36%)
G20	Essential hypertension	126 (20.64%)
N05	Osteoarthritis and allied disorders	122 (20.00%)
N14	Other and unspecified back disorders	121 (19.84%)
R00	General symptoms	108 (17.76%)
C10	Diabetes mellius	106 (17.44%)
**Disease Name and Code**		**Factor 4 diseases prevalence**
C04	Acquired hypothyroidism	27 (44.44%)
N05	Osteoarthritis and allied disorders	21 (34.92%)
N24	Other soft tissue disorder	18 (30.16%)
H06	Acute Bronchitis and Bronchiolitis	17 (28.57%)
R00	General symptoms	16 (26.98%)
G20	Essential hypertension	15 (23.80%)
F46	Cataract	12 (19%)
N09	Other and unspecified joint disorders	12 (19%)
**Disease Name and Code**		**Factor 6 diseases prevalence**
N05	Osteoarthritis and allied disorders	179 (35.19%)
G20	Essential hypertension	171 (33.65%)
N24	Other soft tissue disorder	168 (33.07%)
N09	Other and unspecified joint disorders	132 (25.96%)

© 2022. Longo E et al. Published online at http://www.aginganddisease.org/EN/10.14336/AD.2022.0829

H06	Acute Bronchitis and Bronchiolitis	129 (25.38%)
N14	Other and unspecified back disorders	114 (22.50%)
K19	Other urethral and urinary tract disorders	91 (17.88%)
R00	General symptoms	86 (16.92%)

Supplementary Table 8. Diseases prevalence period 3.

Disease Name and Code		Factor 1 diseases prevalence
H06	Acute Bronchitis and Bronchiolitis	1243 (21.05%)
N24	Other soft tissue disorder	961 (16.27%)
N05	Osteoarthritis and allied disorders	951 (16.10%)
G20	Essential hypertension	902 (15.27%)
N09	Other and unspecified joint disorders	776 (13.14%)
N14	Other and unspecified back disorders	760 (12.87%)
R00	General symptoms	748 (12.67%)
H05	Other acute upper respiratory infections	682 (11.55%)
**Disease Name and Code**		**Factor 2 diseases prevalence**
G20	Essential hypertension	51 (28.33%)
N05	Osteoarthritis and allied disorders	50 (27.77%)
H06	Acute Bronchitis and Bronchiolitis	47 (26.11%)
N24	Other soft tissue disorder	41 (22.77%)
N14	Other and unspecified back disorders	33 (18.33%)
N09	Other and unspecified joint disorders	30 (16.67%)
H05	Other acute upper respiratory infections	29 (16.11%)
F4C	Disorders of conjunctiva	27 (15%)
**Disease Name and Code**		**Factor 3 diseases prevalence**
N05	Osteoarthritis and allied disorders	3 (33%)
N24	Other soft tissue disorder	3 (33%)
H06	Acute Bronchitis and Bronchiolitis	3 (33%)
N09	Other and unspecified joint disorders	2 (22)%
N14	Other and unspecified back disorders	2 (22)%
N21	Peripheral enthesopathies and allied syndromes	2 (22)%
H05	Other acute upper respiratory infections	1 (11%)
E20	Neurotic disorders	1 (11%)
**Disease Name and Code**		**Factor 4 diseases prevalence**
N05	Pruritus and related conditions	48 (24.00%)
H06	Acquired hypothyroidism	46 (23.00%)
N24	Other soft tissue disorder	46 (23.00%)
K19	Other urethral and urinary tract disorders	41 (20.48%)
N09	Other and unspecified joint disorders	39 (19.28%)
C04	Acquired hypothyroidism	36 (18.07%)
N14	Other and unspecified back disorders	56 (27.77%)
**Disease Name and Code**		**Factor 5 diseases prevalence**
G33	Angina Pectoris	93 (46%)
C10	Diabetes Mellitus	50 (25%)
C32	Disorders of liboid metabolism	48 (25%)
G20	Essential hypertension	42 (21%)
H06	Acute Bronchitis and Bronchiolitis	41 (20.19%)
G30	Acute myocardial infarction	35 (17.37%)
**Disease Name and Code**		**Factor 6 diseases prevalence**
C04	Acquired Hypothyroidism	37 (29%)
F50	Disorders of external ear	27 (21%)
G33	Angina Pectoris	27 (21%)
H06	Acute Bronchitis and Bronchiolitis	27 (21%)
H30	Bronchitis unspecifies	27 (21%)

SUPPLEMENTARY DATA

Supplementary Table 9. Diseases prevalence period 4.

Disease Name and Code		Factor 1 diseases prevalence
H06	Acute Bronchitis and Bronchiolitis	124 (29.41%)
N24	Other soft tissue disorder	92 (21.88%)
N09	Other and unspecified joint disorders	86 (20.47%)
N14	Other and unspecified back disorders	85 (20.24%)
N05	Osteoarthritis and allied disorders	82 (19.52%)
K19	Other urethral and urinary tract disorders	74 (17.65%)
H05	Other ccute upper respiratory infections	71 (16.94%)
G20	Essential hypertension	70 (16.70%)
**Disease Name and Code**		**Factor 2diseases prevalence**
N24	Other soft tissue disorder	46 (24.86%)
H06	Acute Bronchitis and Bronchiolitis	45 (24.32%)
N05	Osteoarthritis and allied disorders	40 (21.62%)
N09	Other and unspecified joint disorders	38 (20.54%)
N14	Other and unspecified back disorders	35 (18.91%)
G20	Essential hypertension	31 (16.76%)
R02	Function Gastrointestinal tract disorders	41 (22.22%)
C04	Acquired hypothyroidism	10 (5.56%)
**Disease Name and Code**		**Factor 3 diseases prevalence ****
H33	Asthma	64 (40.00%)
H06	Acute Bronchitis and Bronchiolitis	55 (34.37%)
N24	Other soft tissue disorder	37 (23.12%)
H05	Other acute respiratory infections	29 (18.12%)
R06	Respiratory systems and chest symptoms	27 (16.80%)
G33	Angina pectoris	26 (16.20%)
J10	Diseases of oesophagus	26 (16.20%)
G33	Angina pectoris	26 (16.2)%
**Disease Name and Code**		**Factor 4 diseases prevalence *****
N14	Other and unspecified back disorders	8 (35%)
E20	Neurotic disorders	7 (30%)
R02	Function Gastrointestinal tract disorders	7 (30%)
M18	Pruritus and related conditions	6 (26.09%)
**Disease Name and Code**		**Factor 5 diseases prevalence**
H33	Asthma	4 (100%)
E20	Neurotic disorders	2 (50%)
H06	Acute Bronchitis and Bronchiolitis	2 (50%)

** Note that almost 14% of people suffers from hypertension
*** Note that 18% of people suffers from dermatitis and other eczemas and the same number of people suffers from Psoriasis and similar disorders

Supplementary Table 10. Gender and age composition in all periods before the diagnosis.

Gender composition			
	**CLUSTER 1**	**CLUSTER 2**	**CLUSTER 3**
**MALES**	11875 (35.50%)	11541 (34.50%)	7426 (22.20%)
**FEMALES**	21576 (64.50%)	21910 (65.40%)	26025 (77.80%)
**Age composition**			
**AGE BAND**	**CLUSTER1**	**CLUSTER 2**	**CLUSTER 3**
**<65**	1907 (5.70%)	1840 (5.50%)	723 (2.16%)
**65-75**	4583 (13.70%)	4416 (13.20%)	2823 (8.44%)
**75-85**	14451 (43.20%)	14317 (42.80%)	12912 (38.60%)
**>86**	12511 (37.40%)	12879 (38.50%)	16993 (50.80%)

© 2022. Longo E et al. Published online at http://www.aginganddisease.org/EN/10.14336/AD.2022.0829

SUPPLEMENTARY DATA

Supplementary Table 11. Gender and age composition in period 1

Gender composition			
	CLUSTER 1	CLUSTER 2	CLUSTER 3
MALES	6335 (35.30%)	2084 (35.10%)	167 (23.80%)
FEMALES	11612 (64.70%)	3854 (64.90%)	534 (76.20%)
Age composition			
AGE BAND	CLUSTER 1	CLUSTER 2	CLUSTER 3
<65	998 (5.56%)	333 (5.60%)	25 (3.54%)
65-75	2441 (13.60%)	808 (13.60%)	61 (8.66%)
75-85	7778 (43.34%)	2565 (43.20%)	278 (39.60%)
>86	6730 (37.50%)	2233 (37.60%)	338 (48.20%)

Supplementary Table 12. Gender and age composition in period 2.

Gender composition				
	CLUSTER 1	CLUSTER 2	CLUSTER 3	CLUSTER 5
MALES	4988 (35.20%)	203 (33.30%)	12 (19.70%)	107 (21%)
FEMALES	9182 (64.80%)	407 (66.70%)	49 (80.30%)	401 (79%)
Age composition				
AGE BAND	CLUSTER 1	CLUSTER 2	CLUSTER 3	CLUSTER 5
<65	666 (4.70%)	20 (3.20%)	2 (3.00%)	14 (2.80%)
65-75	1842 (13.00%)	66 (10.90%)	7 (11.80%)	50 (9.90%)
75-85	6235 (44.00%)	254 (41.60%)	26 (42.60%)	201 (39.50%)
>86	5427 (38.30%)	270 (44.30%)	26 (42.60%)	4243 (7.80%)

Supplementary Table 13. Gender and age composition in period 3.

Gender Composition						
	CLUSTER 1	CLUSTER 2	CLUSTER 3	CLUSTER 4	CLUSTER 5	CLUSTER 6
MALES	1990 (33.70%)	1577 (26.70%)	55 (33.20%)	2 (22%)	70 (35%)	39 (30%)
FEMALES	3916 (66.30%)	4329 (73.30%)	112 (66.80%)	7 (78 %)	131 (65%)	92 (71%)
Age composition						
AGE BAND	CLUSTER 1	CLUSTER 2	CLUSTER 3	CLUSTER 4	CLUSTER 5	CLUSTER 6
<65	255 (4.32%)	3 (1.64%)	5 (3.10%)	0 (0%)	3 (1.50%)	3 (2.51%)
65-75	704 (11.92%)	16 (8.86%)	21 (12.40%)	1 (11%)	21 (10.40%)	11 (8.59%)
75-85	2553 (43.22%)	75 (41.7%)	71 (42.30%)	4 (44%)	91 (45.30%)	52 (40.50%)
>86	2393 (40.52%)	86 (47.8%)	70 (42.20%)	4 (44%)	86 (42.80%)	62 (48.40%)

Supplementary Table 14. Gender and age composition in period 4.

Gender composition					
	CLUSTER 1	CLUSTER 2	CLUSTER 3	CLUSTER 4	CLUSTER 5
MALES	122 (29.00%)	47 (25.60%)	55 (29.70%)	39 (24.40%)	6 (28%)
FEMALES	299 (71.00%)	137 (74.40%)	129 (70.30%)	122 (75.60%)	17 (72%)
Age composition					
AGE BAND	CLUSTER 1	CLUSTER 2	CLUSTER 3	CLUSTER 4	CLUSTER 5
<65	11 (2.56%)	3 (1.70%)	3 (1.96%)	1 (2.94%)	0 (0%)
65-75	41 (9.74%)	16 (8.60%)	14 (8.84%)	2 (7.56%)	1 (25%)
75-85	178 (42.30%)	81 (43.8%)	67 (41.50%)	9 (41.50%)	2 (50%)
>86	191 (45.40%)	85 (45.9%)	77 (47.70%)	11 (48.00%)	1 (25%)

© 2022. Longo E et al. Published online at http://www.aginganddisease.org/EN/10.14336/AD.2022.0829

SUPPLEMENTARY DATA

Supplementary Table 15. Number of people in each cluster.

PERIOD	CLUSTER	NUMBER	%	TOTAL NUMBER
1	1	17947	66.55%	26968
	2	5938	22.02%	
	3	701	2.60%	
2	1	14171	69.70%	20332
	2	610	3.00%	
	3	61	0.30%	
	4	508	2.50%	
3	1	5906	41.10%	14369
	2	180	1.25%	
	3	167	1.16%	
	4	9	0.06%	
	5	201	1.40%	
	6	129	0.90%	
4	1	421	5.50%	7660
	2	184	2.40%	
	3	161	2.10%	
	4	23	0.30%	
	5	4	0.05%	

© 2022. Longo E et al. Published online at http://www.aginganddisease.org/EN/10.14336/AD.2022.0829
